# Thermomechanical Behavior of Poly(3-hexylthiophene) Thin Films on the Water Surface

**DOI:** 10.1021/acsomega.2c01451

**Published:** 2022-06-01

**Authors:** Boo Soo Ma, Jin-Woo Lee, Hyeonjung Park, Bumjoon J. Kim, Taek-Soo Kim

**Affiliations:** † Department of Mechanical Engineering, Korea Advanced Institute of Science and Technology (KAIST), Daejeon 34141, Republic of Korea; ‡ Department of Chemical and Biomolecular Engineering, Korea Advanced Institute of Science and Technology (KAIST), Daejeon 34141, Republic of Korea

## Abstract

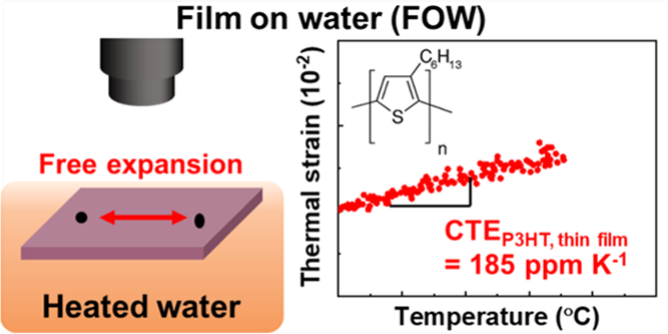

The thermomechanical behavior of a conjugated polymer (CP) in a thin film state has rarely been studied despite the importance of understanding the polymer morphologies and optimizing the thermal processes of organic semiconductors. Moreover, the seamless integration of multilayers without mechanical failures in CP-based electronic devices is crucial for determining their operational stability. Large differences in the coefficients of thermal expansion (CTEs) between the multilayers can cause serious degradation of devices under thermal stress. In this study, we measure the intrinsic thermomechanical properties of poly(3-hexylthiophene) (P3HT) thin films in a pseudo-freestanding state on the water surface. The as-cast P3HT thin films exhibited a large thermal shrinkage (−1001 ppm K^–1^) during heating on the water surface. Morphological analyses revealed that the thermal shrinkage of the polymer films was caused by the rearrangement of the polymer chain networks accompanied by crystallization, thus indicating that preheating the polymer films is essential for estimating their intrinsic CTE values. Moreover, the rigidity of the substrate significantly influences the thermomechanical behavior of the polymer films. The polymer films that were preheated on the glass substrate showed nonlinear thermal expansion due to the substrate constraint inhibiting sufficient relaxation of the polymer chains. In comparison, a linear expansion behavior is observed after preheating the films on the water surface, exhibiting a consistent CTE value (185 ppm K^–1^) regardless of the number of thermal strain measurements. Thus, this work provides a direct method for measuring in-plane CTE values and an in-depth understanding of the thermomechanical behaviors of CP thin films to design thermomechanically reliable organic semiconductors.

## Introduction

Organic semiconductors (e.g., organic photovoltaics (OPVs) and organic-field-effect transistors (OFETs)) are generally composed of various functional layers such as substrates, transport layers, and conjugated polymer (CP) thin films.^1−5^ The temperature sensitivity of organic materials has been considered as a major limitation, rendering organic devices thermally unstable as compared to inorganic semiconductors.^6−13^ For example, thermal annealing is commonly performed to improve the electrical performance of organic semiconductors, while excessive annealing may cause device failure due to phase separation or residual thermal stress.^14,15^ In particular, temperature changes during device fabrication and operation inevitably exert thermal stress on these stacked thin films. This thermal stress is directly proportional to the coefficient of thermal expansion (CTE) mismatch between the stacked films with different materials and thicknesses.^16−19^ Consequently, thermomechanical failures such as cracking or delamination of CP thin films occur under the temperature variation applied to the films.^20−27^ For instance, exposing organic solar cells to sun light can result in the increase of device temperature up to 85 °C, which typically causes significant degradation of the power conversion efficiency of the organic solar cells.^21,28−30^ CP thin films are often exposed to elevated temperature in device fabrication processes.^31−33^ It is known that mechanical failures of polymer thin films such as delamination^17,18,28,34,35^ and cracking^36−41^ may occur after the thermal annealing process, which is a type of thermal treatment that heats the device to a specific temperature for a certain period of time to improve electrical performance by enhancing the ordering of polymer chains.^42−44^ Thus, understanding and quantification of the thermomechanical behavior of CP thin films are critical to manufacture organic semiconductors with excellent electrical performance and superior thermomechanical reliability at the same time. However, measuring the CTE of CP thin films with a thickness of tens of nanometers is challenging as compared to that for substrates with a thickness of several micrometers because handling such extremely thin film samples is significantly difficult.

In previous reports, ellipsometry has been widely used to determine the CTE of polymeric thin films.^26,45−51^ Using ellipsometry, the temperature-induced thickness change is measured by observing the characteristics of the polarized light reflected from a thin film supported on a rigid substrate. However, the substrate constraint complicates the measurement of the intrinsic thermomechanical properties of polymer thin films.^52−54^ Change in the thermal strain of the thin film in the out-of-plane (OOP) direction per unit temperature is not equal to the intrinsic CTE of the thin film, owing to the plane stress state in the film–substrate bilayer. To calculate the CTE of thin films using the ellipsometry method, additional mechanical properties such as Young’s modulus and Poisson’s ratio of thin films and the substrate should be assumed, resulting in a certain inaccuracy in the measurement of the CTE of the film.^45,46,55,56^ In particular, the mechanical properties of the rigid substrate greatly influence the thermomechanical behavior of the polymer films when the temperature increases. Recently, we developed a method to directly measure the thermal strain of thin films on water surfaces.^57^ It is possible to obtain the intrinsic CTE value of thin films by measuring the thermal strain of the films on the water surface via the digital image correlation (DIC) technique that excludes the constraining influence of rigid substrates.

In this study, we investigate the thermomechanical behaviors of CP thin films by floating them on frictionless water surfaces. We choose regioregular poly(3-hexylthiophene) (P3HT), a widely studied conjugated polymer, as a model polymer in this study. Whereas most polymers generally expand upon heating, the as-cast non-preheated P3HT thin films exhibit a large thermal shrinkage (−1001 ppm K^–1^). Morphological analyses reveal that the thermal shrinkage of CP thin films is induced by crystallization of polymer chains as many chains are not sufficiently arranged and crystallized during the spin-coating process.^58^ Considering this, P3HT thin films are also preheated on the glass substrate to allow sufficient arrangement of P3HTs prior to the CTE measurement. However, P3HT films preheated on glass exhibit nonlinear and negative thermal expansion caused by the confinement of the rigid substrate along the in-plane (IP) direction during the preheating process. In contrast, when P3HT films are preheated on water surfaces, we observe repetitive linear expansion of P3HT thin films, which affords a constant CTE value of 185 ppm K^–1^. This is because the polymer chains can be sufficiently rearranged and crystallized on water without the substrate constraints. Therefore, we conclude that preheating CP thin films on the water surface is an essential process for precisely investigating the thermomechanical properties without thermal history.

## Experimental Section

### Materials

Regioregular P3HT was purchased from RIEKE METALS (4002-EE). Poly(styrenesulfonate) (PSS) and chlorobenzene (CB) were purchased from Sigma-Aldrich. P3HT was dissolved in CB (20 mg mL^–1^), and this solution was spin-coated onto the PSS-coated glass substrate. Preheating of P3HT thin films on PSS/glass was performed by placing the samples directly on a hot plate with different temperatures (50, 100, 150 °C) in 1 h after spin-coating. The preheating of P3HT thin films on water surfaces was performed after transferring the films on water surfaces at 75 °C in 1 h. All of the P3HT films on PSS/glass were coated and heated under laboratory air conditions (∼25 °C, ∼30% relative humidity) and then dried for at least 72 h in a vacuum desiccator.

### Characterization

The number-average molecular weight (*M*_n_) and polydispersity index (*Đ*) of the P3HT polymer were measured by size exclusion chromatography (SEC) relative to polystyrene standards using tetrahydrofuran (THF) as the eluent, with an Agilent GPC 1200 series instrument equipped with a refractive index detector. The thermal properties of the P3HT polymer were collected using a TA Instruments DSC 25 from the second heating/cooling cycle from 20 to 250 °C at a rate of 10 °C min^–1^.

### Thermal Strain Measurements

Before the measurement, a femtosecond laser was used to cut the coated films into a 5 mm by 25 mm rectangular shape to remove edge effects. Graphite particles were sprayed on P3HT thin films as markers to measure thermal strains. By submerging P3HT thin film/PSS/glass samples, the films were floated on the deionized water surface as PSS dissolved in water. Subsequently, one end of the floated film was fixed to the polydimethylsiloxane (PDMS)-coated aluminum grip by van der Waals adhesion. The thermal strain values were calculated based on the film surface images captured by a charge-coupled device (CCD) camera (Manta, G-504, Germany). The temperature of water surfaces was recorded using a digital multimeter (Fluke, 54-II-B). Water surfaces were heated up to 75 °C, not the boiling point of water (∼100 °C), to prevent air bubbles that inhibit pattern tracking by shading and damage the films. All of the measurements were performed under laboratory air conditions.

### GIXS Measurement

GIXS measurement was performed at the beamline 9A at the Pohang Accelerator Laboratory, Korea. The incidence angles were about 0.12–0.14° for penetrating the thin films completely. Scherrer equation was used to calculate the *L*_c_ of polymer films.
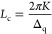
*K* and Δ_q_ are shape factor (0.9) and full width at half-maximum of scattered peaks, respectively.

## Results and Discussion

Regioregular P3HT (regioregularity = 91%) with the *M*_n_ of 22 kg mol^–1^ and *Đ* of 2.5 was used in this study. These values were estimated by SEC using THF as the eluent (Figure S1). The P3HT polymer dissolved in CB solution was used to fabricate CP thin films with a thickness of ∼120 nm. P3HT films were spin-coated onto a PSS/glass substrate. PSS was used as a sacrificial layer to float the thin films on the water surfaces. Fine graphite particles were sprayed onto P3HT films for the thermal strain measurement before transferring them to the water surface. P3HT/PSS/glass samples were then carefully immersed to float the P3HT films on the water surface. The detailed characterization and preparation procedure of P3HT thin film samples are described in the Experimental Section.

**Figure 1:**
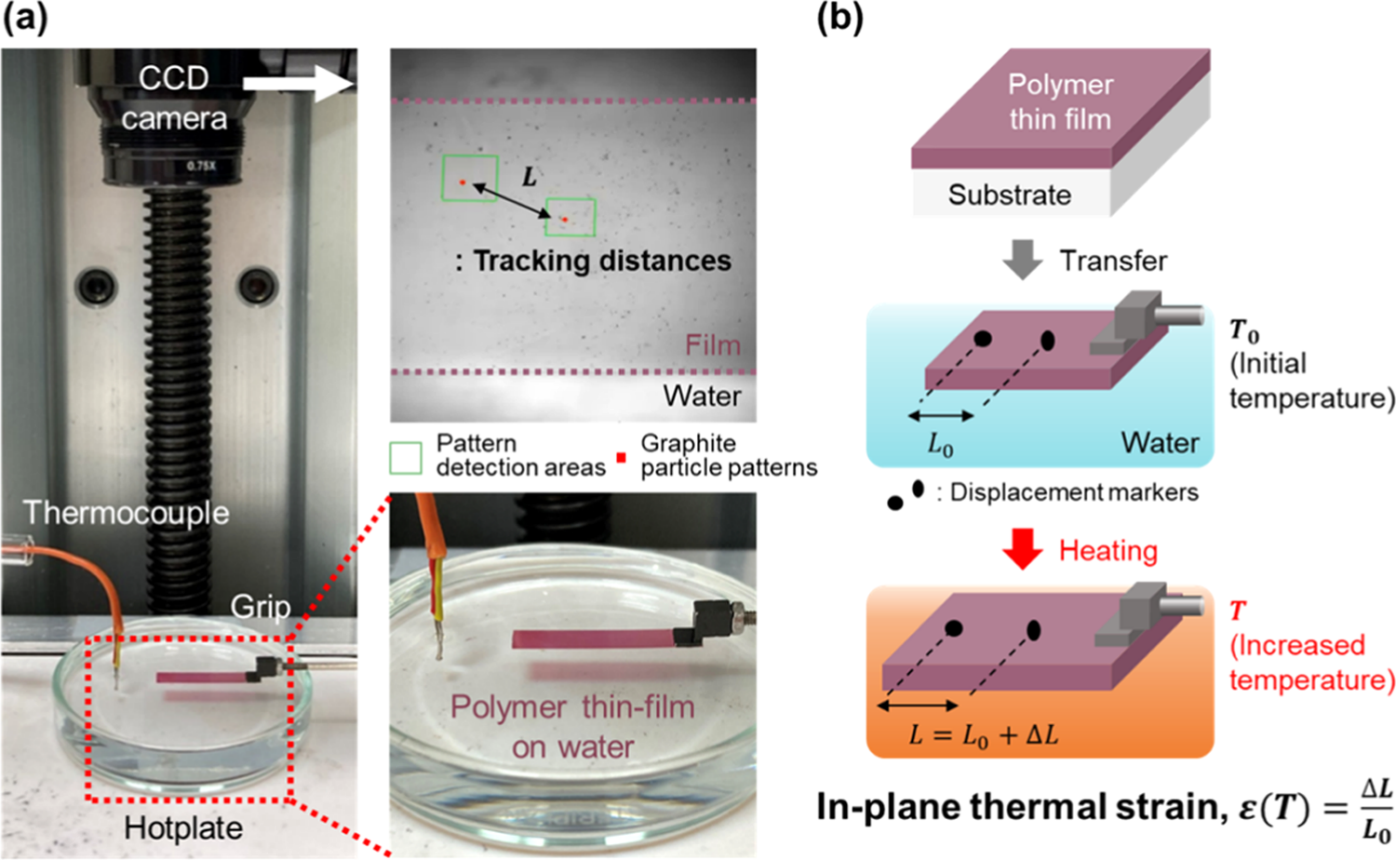
(a) Experimental setup and (b) principle of thermal strain measurement for conjugated polymer thin films.

IP thermal strains of polymer thin films were measured as illustrated in Figure 1a. One end of the P3HT thin film on the water surface was fixed using an aluminum grip. The water surface allows unobstructed deformation of P3HT films and acts as a heater, thereby enabling us to avoid the constraints of rigid substrates. In addition, thermal deformation of P3HT films was induced by heating water. The film surface images and temperature values of the water surface were recorded simultaneously while heating the water surface at a constant heating rate of ∼17 K min^–1^ using a hot plate (Figure S2). Rectangular P3HT thin films were used in the experiment. The film surface images were recorded excluding the gripped region to avoid the confinement effect of the grip on the film. The water surface was heated from 25 to 75 °C to prevent the formation of bubbles as the water boiled. To measure the thermal strain, fine graphite particles were sprayed onto P3HT thin films as markers. The location of the particles was tracked via the two-dimensional DIC technique based on the surface images of P3HT thin films. We calculated the thermal strain values of P3HT thin films as the ratio of the change in distance to the initial distance between the two particles from the location data of the particles (Figure 1b). Based on this principle, we measured IP thermal strains of P3HT thin films directly. The changes in film thickness before and after the test were measured to determine the thermal strain of the films in the OOP direction. The detailed procedure for measuring the thermal strains of P3HT thin films is described in the Experimental Section.

**Figure 2:**
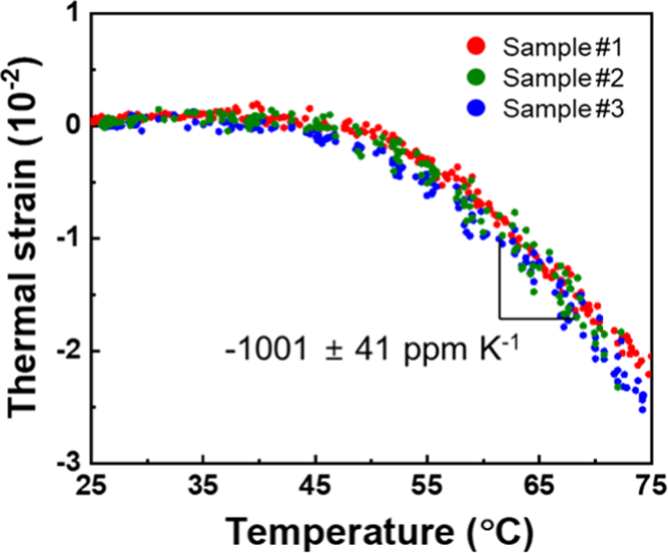
Thermal strain of as-cast P3HT thin films as a function of heating temperature.

The thermal strain of the as-cast P3HT thin films as a function of temperature is plotted in Figure 2. Surface images of the as-cast P3HT film captured in the actual experiment are shown in Figure S3. Notably, heating of the as-cast P3HT thin films induced nonlinear thermal shrinkage of the thin films. For example, the as-cast P3HT films start to shrink after 50 °C with a rate of −1001 ± 41 ppm K^–1^ and they shrank by −2.5% at 75 °C. The thermal shrinkage occurred isotropically in the IP direction and was confirmed repeatedly for different P3HT/PSS/glass samples. For example, the thermal shrinkages of P3HT thin films in three different directions were observed, and there was no difference in their thermal strain values depending on the direction (Figure S4). The shrinkage of the as-cast films after 50 °C can be attributed to the crystallization of P3HT over their glass-transition temperature (*T*_g_), especially owing to the side-chain rearrangement of P3HT.^59−63^ In this case, thermal shrinkage due to the crystallization of polymers is dominant as compared to their thermal expansion, and thus, negative thermal strains are observed for the as-cast films.

**Figure 3:**
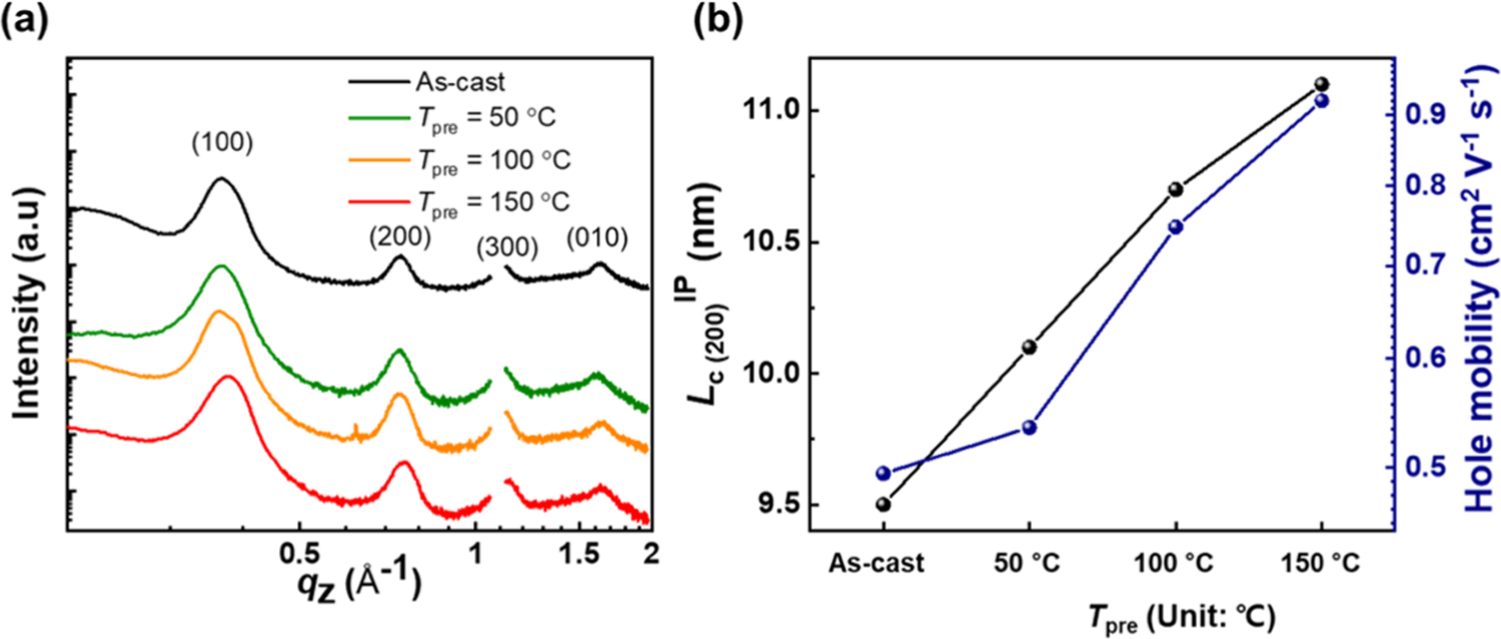
(a) GIXS line-cut profiles in the OOP direction and (b*) L*_c_ values of the OOP (200) peaks and OFET hole mobilities of P3HT films depending on the preheating conditions.

To investigate the reasons for the thermal shrinkage of the as-cast films, the crystalline structure of the films was measured using a grazing incidence wide-angle X-ray scattering (GIXS) measurement. In this analysis, as-cast and preheated P3HT thin films at different preheating temperatures (50, 100, and 150 °C) were compared. The detailed procedure for the X-ray analysis is described in the Experimental Section. Line-cut profiles of the GIXS analysis for P3HT thin films are plotted in Figures 3a and S7. All of the films show distinct high-order (*h*00) scatterings in both the IP and OOP directions, indicative of the presence of crystalline structures with lamellar interactions of P3HT in the thin film. In addition, broad halos in a *q* range of 1.0–2.0 Å^–1^ are observed in both the IP and OOP directions, which suggests the coexistence of amorphous regions in the thin films.^64^ Notably, there are differences in the OOP (*h*00) peaks depending on the preheating temperature (*T*_pre_), showing narrower distributions with elevated *T*_pre_. For quantitative analysis, we estimated the coherence length (*L*_c_) values of the (200) peaks in the OOP direction based on the Scherrer equation (Figure S8).^65,66^ The *L*_c_ of P3HT films preheated on PSS/glass increased with higher *T*_pre_, suggesting that the crystalline domain sizes of P3HT became larger and that the polymer chains were arranged into more-ordered fashion at higher temperatures. For instance, the as-cast P3HT film exhibited *L*_c_ = 9.5 nm, and it increased to 10.1 nm at *T*_pre_ = 50 °C, and to 11.1 nm at *T*_pre_ = 150 °C. The increasing *L*_c_ values proportional to *T*_pre_ agree well with previous reports,^67,68^ and the trend suggests that the preheating process develops larger crystalline domains by the rearrangement of disordered polymer chains in P3HT thin films. The increased crystallinities of the films upon heating were further supported by charge mobility measurements in the organic field-effect transistor (OFET) (Figure S9 and Table S1). The increased heating temperature linearly increased the saturated hole mobility (μ_sat_^OFET^) of P3HT films. For example, μ_sat_^OFET^ increased from 4.95 × 10^–5^ cm^2^ V^–1^ s^–1^ for the as-cast film to 7.47 × 10^–5^ cm^2^ V^–1^ s^–1^ upon heating to 100 °C, and to 9.22 × 10^–5^ cm^2^ V^–1^ s^–1^ upon heating to 150 °C. This suggests that the crystalline domains in P3HT thin films increased gradually by increasing *T*_pre_ from 50 to 150 °C, which is in agreement with the GIXS results. Thus, the thermal shrinkage behavior observed in the as-cast film was mainly due to the increased packing density of polymer chains accompanied by polymer rearrangement and crystallization. Therefore, sufficient crystallization of the polymers should be preceded before thermal strain measurement to precisely measure the CTE values of P3HT films upon thermal expansion.

**Figure 4:**
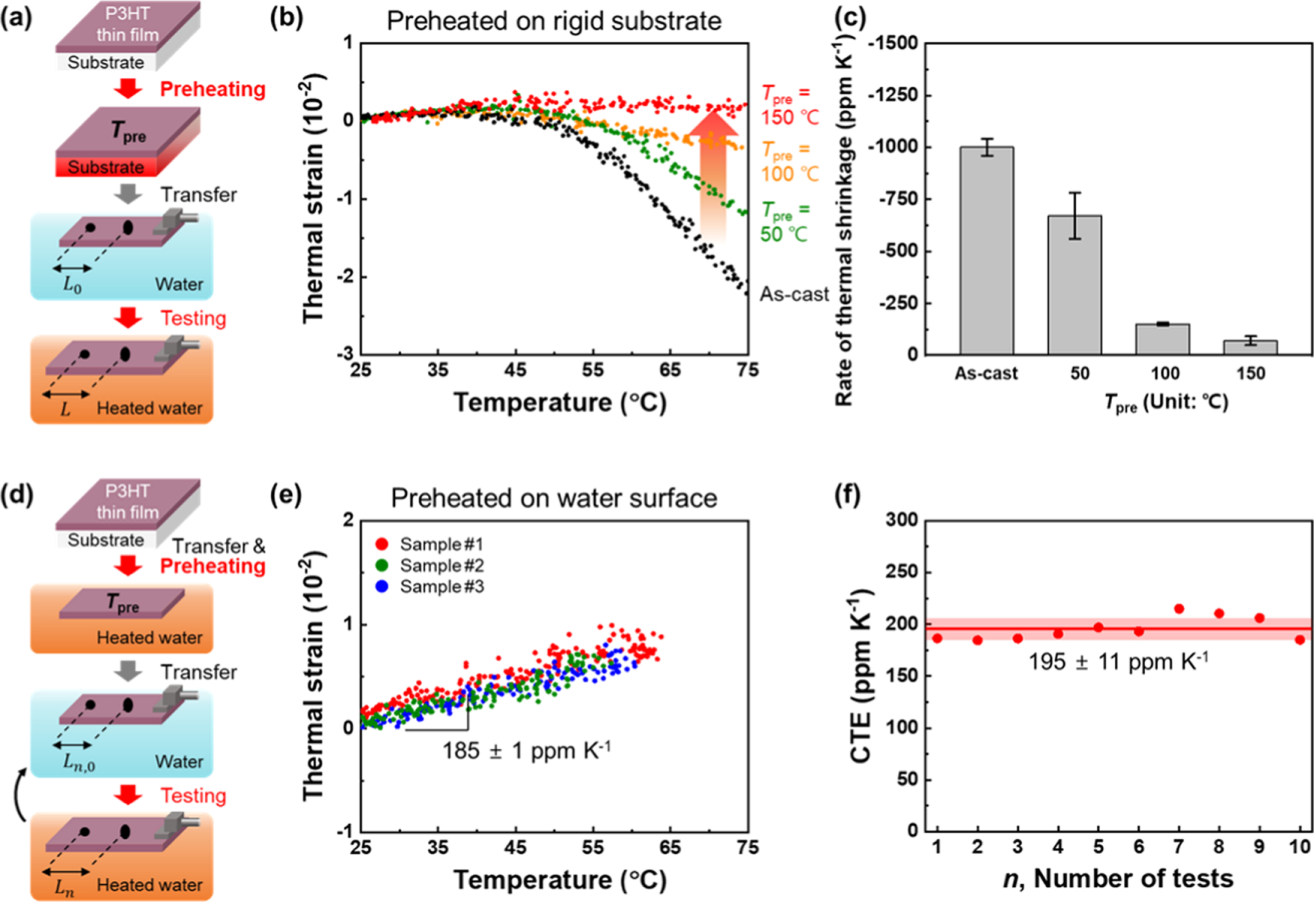
(a) Schematic illustration of the “preheating on solid substrate” process. (b) Thermal strain and (c) rate of thermal shrinkage of P3HT thin films with different preheating temperatures. (d) Schematic illustration of the “preheating on water surface” process. (e) Thermal strain of P3HT thin films after preheating on water. (f) CTE of P3HT thin films with different testing cycles.

The existing literature also indicates that unarranged polymer chains such as loosely packed crystals/amorphous chains are formed in the as-cast state, owing to the fast solidification of the polymers during the solution process.^46,69,70^ To minimize these disordered segments and ensure sufficient crystallization of P3HT polymers, the as-cast P3HT films on rigid substrates were preheated at different temperatures. Subsequently, their thermal strain values were analyzed using the same method as described in the previous section (Figure 4). P3HT/PSS/glass samples were heated for 1 h at three different *T*_pre_—50, 100, and 150 °C—as high as possible without degrading the sacrificial layer. Consequently, the thermal shrinkage of P3HT films gradually decreased as *T*_pre_ increased (Figure 4b). The rates of thermal shrinkage decreased from −1001 ± 41 ppm K^–1^ to −672 ± 111, −151 ± 7, and −77.2 ± 38.9 ppm K^–1^ as *T*_pre_ increased to 50, 100, and 150 °C, respectively (Figure 4c). This suggests that the preheating of the films in the rigid substrates alleviates their shrinkage during the CTE measurement, and the extent of alleviation differs according to the *T*_pre_ values. However, linear thermal expansion was not observed even when *T*_pre_ was increased to 150 °C. This implies that the rigid substrates inhibited the complete relaxation of the polymer chains, resulting in the residual remaining loose chains in the polymer films. That is, nonlinear changes in the thermal strain of the preheated films still appeared because the loosely packed crystals/amorphous chains in the polymer films were not completely removed. Also, this can be attributed to significant thermal mismatches between the glass and P3HT films in terms of their CTE values.

Considering these points, the as-cast P3HT thin films were preheated after being transferred to water surfaces to investigate whether the nonlinear thermal expansion was due to the remaining loose chains after preheating the films on the rigid substrate. The thermal strains of the preheated P3HT films were measured (Figure 4d). In this case, the films can be deformed freely on the water surfaces without constraints from the substrate. Interestingly, the linear expansion behavior of the films was clearly observed in P3HT thin films preheated on water, which is in stark contrast to the results for the as-cast and preheated P3HT thin films on rigid substrates (Figure 4e). In fact, P3HT thin films preheated on the glass substrate exhibited a greater thickness reduction (−11 to −24%) than the films preheated on water (−7%) (Figure S6). This implies that the rigid substrate inhibited polymer chain relaxation along the IP direction rather than the OOP direction, while the as-cast films were deformed freely on water surfaces. Consequently, the CTE of P3HT thin films was 185 ± 1 ppm K^–1^. This CTE value is in good agreement with previous reports on thicker P3HT.^71,72^ To the best of our knowledge, the quantitative CTE value of ultrathin semiconducting films in the IP direction without substrate constraint is measured for the first time in this study. The linear behavior of P3HT films was repeatedly observed even when the test was performed up to 10 times (Figure 4f). Thus, this linear expansion behavior implies that the polymer networks in P3HT thin films were sufficiently rearranged and crystallized by preheating the films without any rigid substrates. This result indicates that preheating of the CP thin films on water surfaces is important for estimating their exact CTE values, excluding the effects of thermal shrinkage from polymer chain relaxations. Moreover, the thermomechanical behavior change can provide a better insight into polymer morphologies in the thin film state. However, it has been reported that the polymer thin films from the polymer solution can have a completely equilibrated morphology when they annealed to a high temperature exceeding the melting point of the polymer crystal. Nevertheless, since the boiling point of water is quite lower than the melting point of P3HT (214.6 °C), further study is necessary for a nonvolatile, water-like liquid platform with a boiling point higher than that of water and the target polymer.

**Figure 5:**
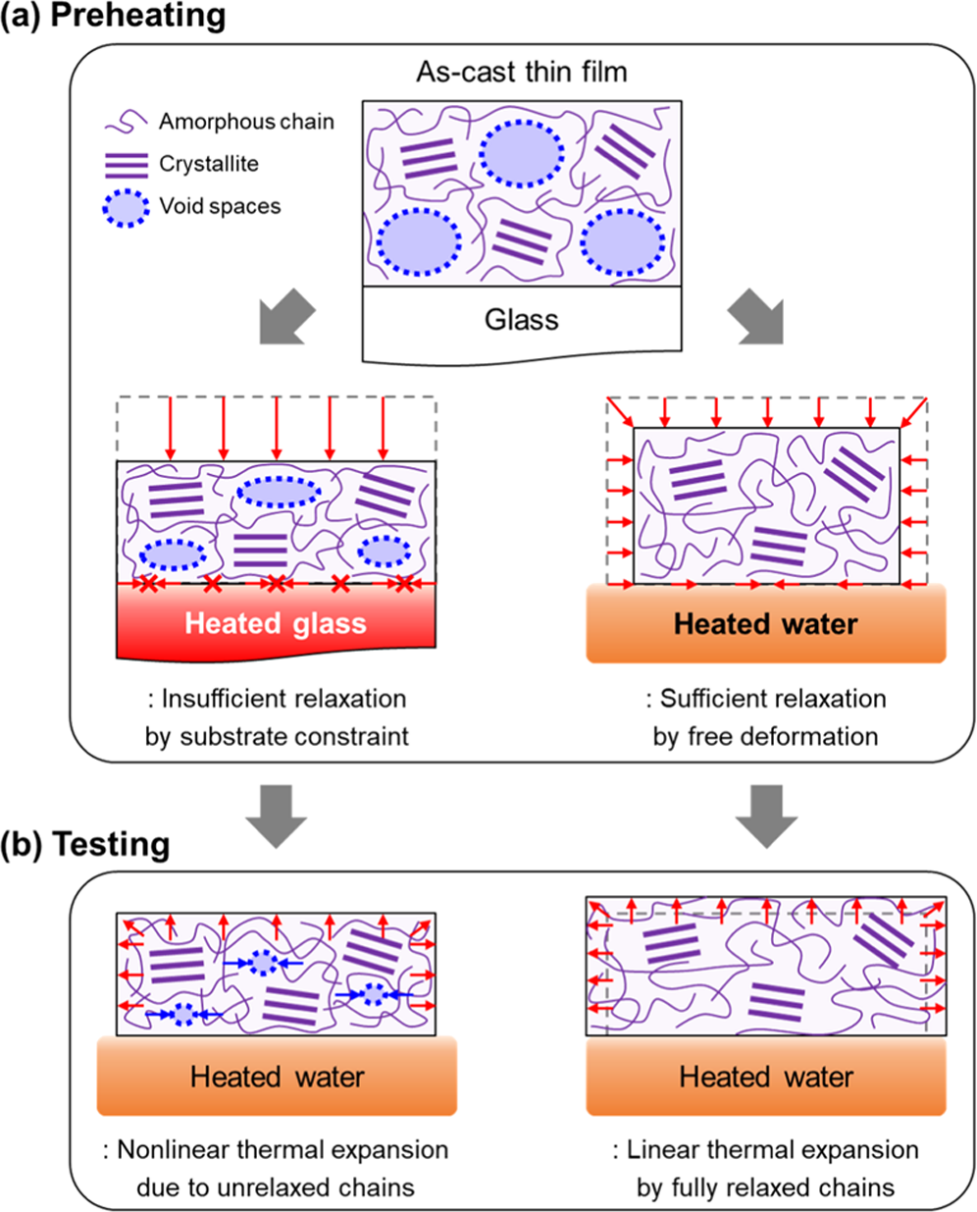
Schematic illustrations of morphology changes of P3HT thin films in (a) preheating and (b) testing procedures.

Conclusively, we suggest the mechanisms of the thermal shrinkage behavior of P3HT thin films under different preheating conditions. As shown in Figure 5, when the P3HT solution is spin-coated on the substrate, the dissolved polymer chains form a large free volume with incomplete crystals. This is because the P3HT chains are not sufficiently arranged owing to fast solidification.^58,73−76^ Preheating of the films on rigid substrates can arrange the polymer chains and grow crystal sizes of P3HT, while the films still represent thermal shrinkage when they are heated on the water surface. This is attributed to the fact that the polymer chains are constrained from the substrates that hinder sufficient rearrangement of P3HT chains in the IP direction. Alternatively, if the as-cast films are preheated on water without the substrate constraint, the polymer chains can be rearranged freely along the IP direction. Thus, it is possible to measure the CTE of polymer thin films from this stabilized state. Therefore, we highlight that preheating the CP films on water is important for estimating the intrinsic thermomechanical behavior of the CP films, such as their precise CTE values, to induce sufficient rearrangement of the CP chains in both the IP and OOP directions.

## Conclusions

In this study, we investigated thermomechanical behaviors of regioregular P3HT thin films in a pseudo-freestanding state on the water surface. The as-cast P3HT films showed nonlinear thermal shrinkage behaviors owing to the relaxation of polymer chains with nominal crystallization of P3HT, as demonstrated by X-ray analysis. The extent of thermal shrinkage decreased with preheating of P3HT films on rigid substrates. However, P3HT films preheated even at 150 °C exhibited nonlinear thermal expansion. This is because the constraint from the rigid substrates inhibits sufficient rearrangement of P3HT chains. When the films were preheated on the water surface, all P3HT films exhibited a distinct linear thermal expansion with a CTE of 185 ppm K^–1^, which was confirmed with repeated tests. The water surface allows the polymer film to be rearranged freely, as opposed to rigid substrates. Moreover, we report the CTE measurement of CPs on water surfaces for the first time, demonstrating a new way to investigate their thermomechanical behaviors. Therefore, preheating CP films on water surfaces is necessary to obtain their exact CTE values. This study contributes to the current understanding of this research area, as the measured thermomechanical properties of CP thin films can be utilized for understanding the morphology of polymer chain networks, such as the effect of substrate type on the polymer film during the heating process. Moreover, we report the CTE measurement of CPs on water surfaces for the first time, demonstrating a new way to investigate their thermomechanical behaviors.

## Supporting Information Available

SEC chromatogram of the P3HT polymer (Figure S1); heating rate of water surfaces in thermal strain measurement (Figure S2); film surface images of as-cast P3HT thin films at different temperatures (Figure S3); (a) surface images and (b) thermal strain of as-cast P3HT thin films by arbitrary directions (Figure S4); DSC thermograms of the P3HT polymer (Figure S5); film thickness of preheated P3HT thin films with different conditions (Figure S6); GIXS line-cut profiles in the in-plane direction of preheated P3HT thin films with different preheating conditions (Figure S7); coherence lengths based on GIXS line-cut profiles (a) in-plane and (b) out-of-plane of preheated P3HT thin films with different preheating conditions (Figure S8); OFET transfer curves of P3HT thin films depending on their annealing temperatures (Figure S9); and OFET parameters estimated from the transfer curves (Table S1) (PDF)
