# Supplementary material for: Thermomechanical Behavior of Poly(3-hexylthiophene) Thin Films on the Water Surface
Source: ACS Omega. 2022 Jun 1;7(23):19706–13. doi: 10.1021/acsomega.2c01451 (PMC9202286; doi:10.1021/acsomega.2c01451)
Supplement: Supplementary file 1 — ao2c01451_si_001.pdf [file ao2c01451_si_001.pdf]

Supporting Information

Thermo-mechanical Behavior of Poly(3-hexylthiophene) Thin Film on Water Surface

Boo Soo Ma,[†, ‡] *Jin-Woo Lee,*[†, §] *Hyeonjung Park,* [§] *Bumjoon J. Kim,*[*, §] *and Taek-Soo Kim*[*,‡]

[‡]Department of Mechanical Engineering, Korea Advanced Institute of Science and Technology (KAIST), Daejeon, 34141, Republic of Korea

[§]Department of Chemical and Biomolecular Engineering, Korea Advanced Institute of Science and Technology (KAIST), Daejeon, 34141, Republic of Korea

[*](T.-S. Kim) E-mail: tskim1@kaist.ac.kr

[*](B. J. Kim) E-mail: bumjoonkim@kaist.ac.kr

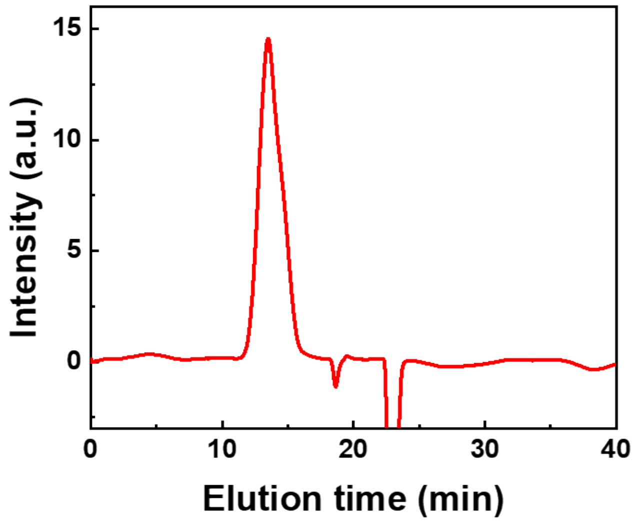

Figure S1. SEC chromatogram of P3HT polymer.

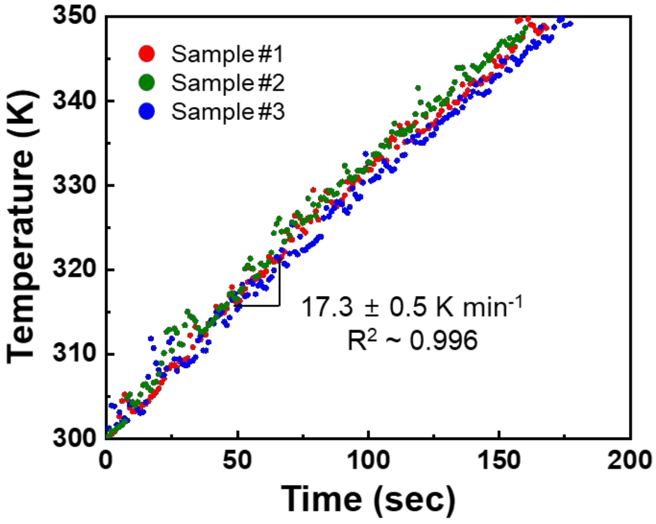

Figure S2. Heating rate of water surfaces in thermal strain measurement.

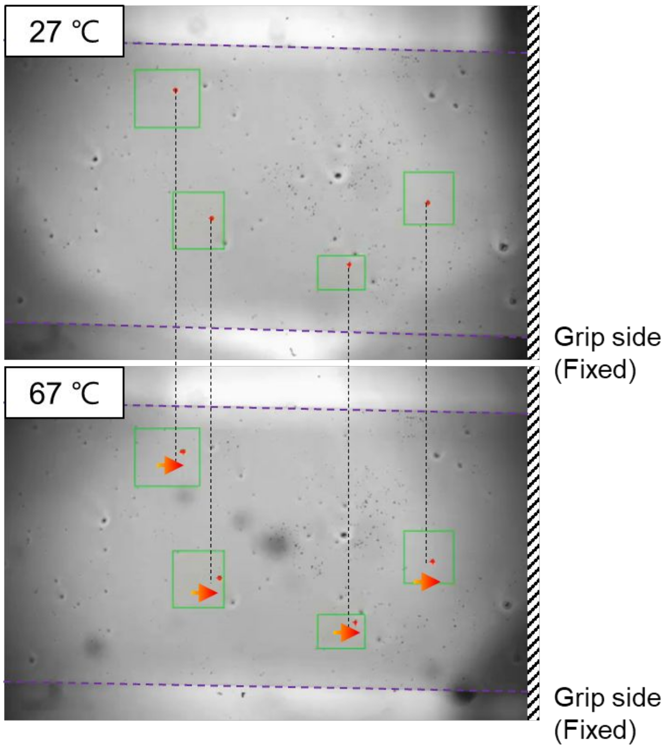

Figure S3. Film surface images of as-cast P3HT thin films at different temperatures.

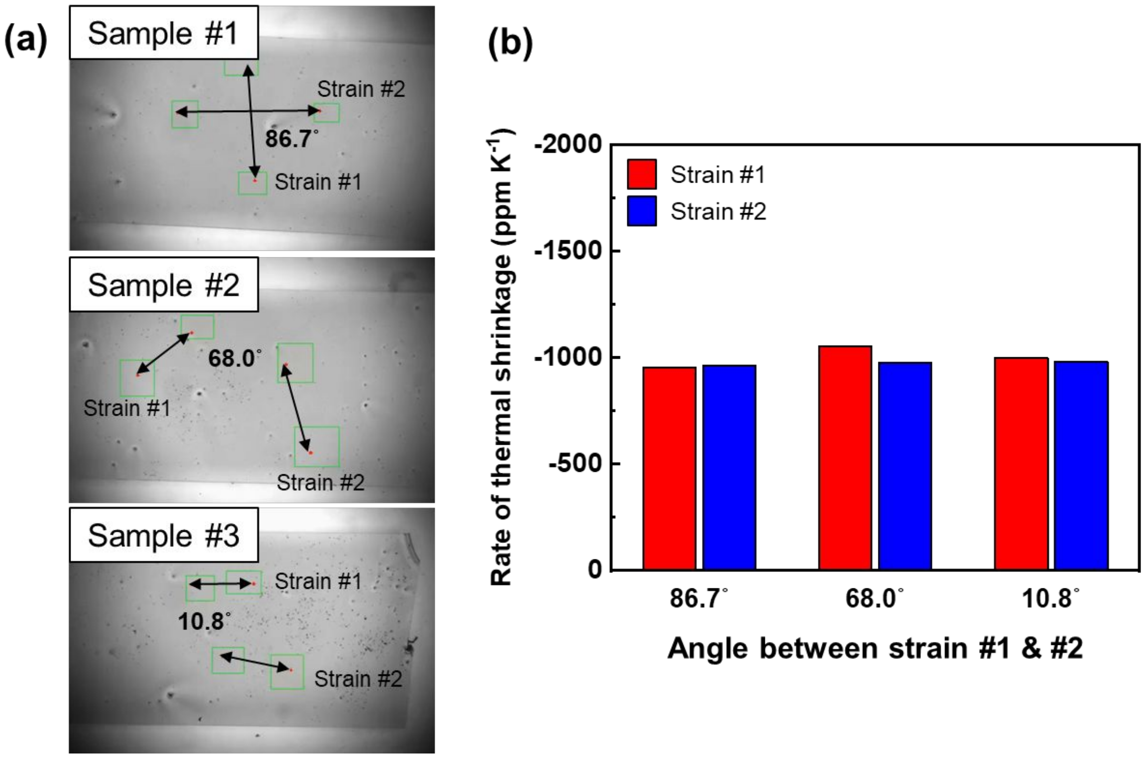

Figure S4. (a) Surface images and (b) thermal strain of as-cast P3HT thin films by arbitrary directions.

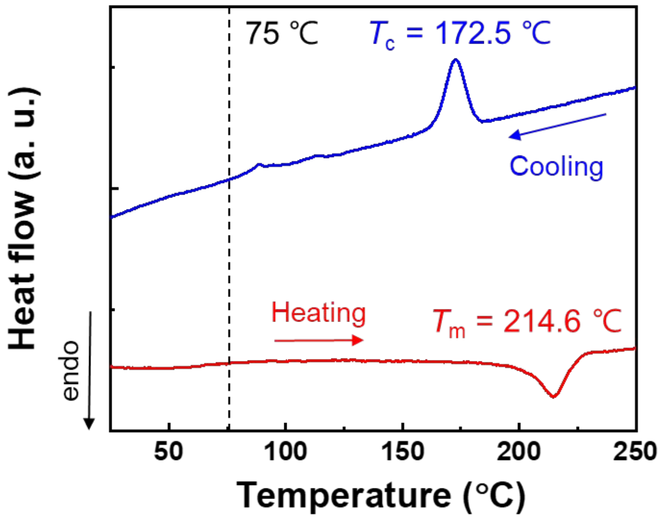

Figure S5. DSC thermograms of P3HT polymer.

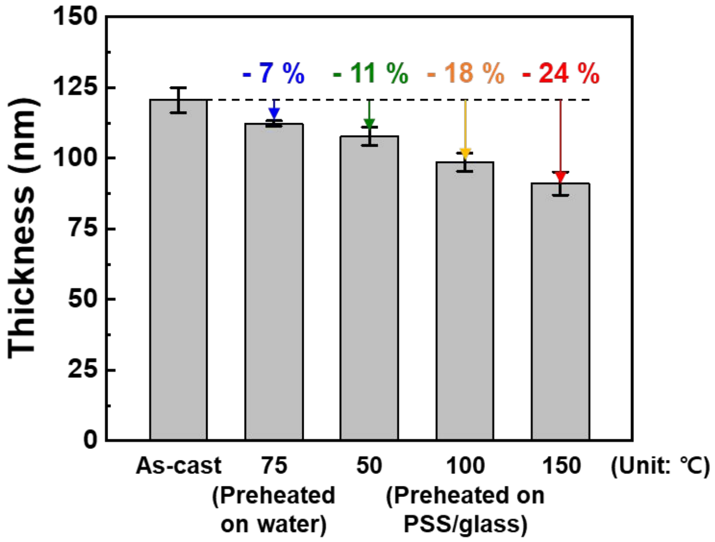

Figure S6. Film thickness of preheated P3HT thin films with different conditions.

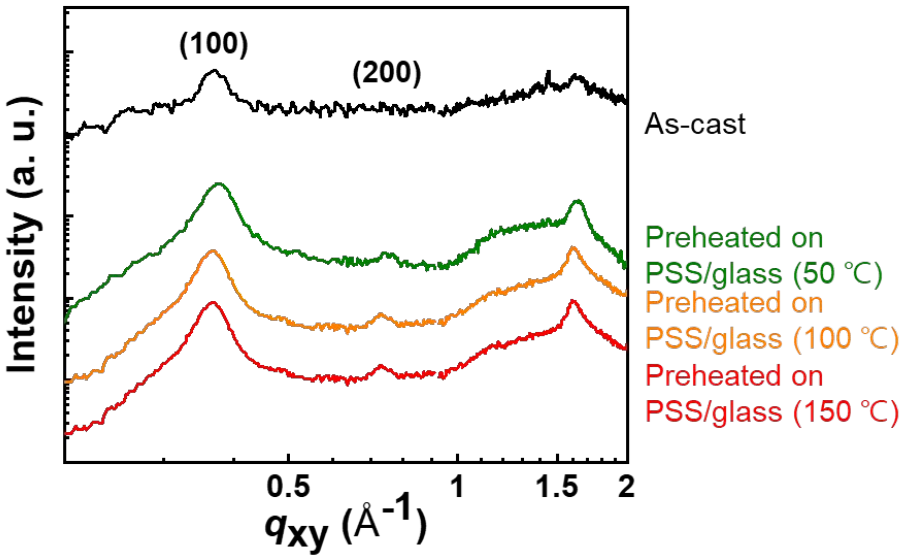

Figure S7. GIXS line-cut profiles in the in-plane direction of preheated P3HT thin films with different preheating conditions.

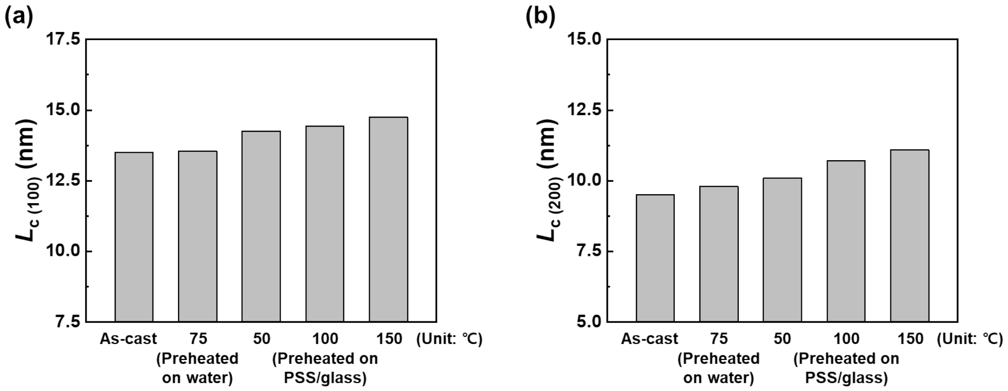

Figure S8. Coherence lengths based on GIXS line-cut profiles (a) in-plane and (b) out-of-plane of preheated P3HT thin films with different preheating conditions.

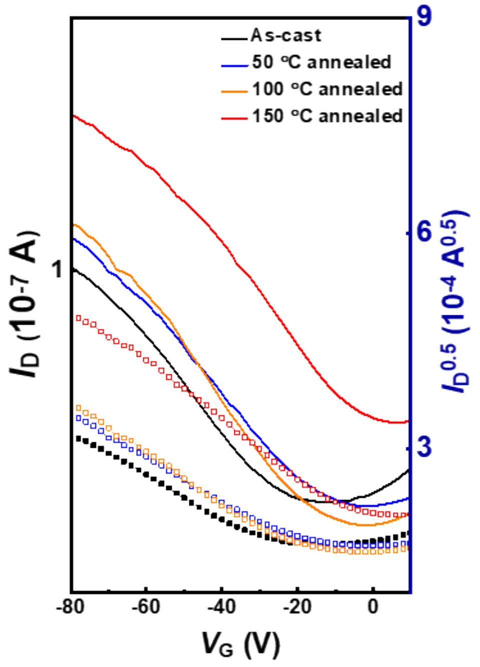

Figure S9. OFET transfer curves of the P3HT thin-films depending on their annealing temperatures.

Table S1. OFET parameters estimated from the transfer curves.

**Temperature (^{0}C)**	μ_{sat}^{OFET} **(cm^2 V^{-1} s^{-1})** [a]	V_{th} **(V)**[A]	$I_{on}\, I_{off}^{-1}$
As cast	4.95×10^{-5}	20.7	$> 10^0$
50	5.35×10^{-5}	25.1	$> 10^1$
100	7.47×10^{-5}	14.4	$> 10^1$
150	9.22×10^{-5}	36.5	$> 10^1$
